# Re-starting anticoagulation and antiplatelets after gastrointestinal bleeding: A systematic review

**DOI:** 10.12688/f1000research.135132.1

**Published:** 2023-07-10

**Authors:** Ethan Slouha, Haley Jensen, Hope Fozo, Rhea Raj, Sneha Thomas, Vasavi Gorantla

**Affiliations:** 1 St George's University School of Medicine, Saint George's, Saint George, Grenada; 2 Department of Internal Medicine, University of Maryland Medical Center, Baltimore, Maryland, USA

**Keywords:** Anticoagulants, Restarting Anticoagulants, GI bleeds, GI bleeding, Resuming Anticoagulants, Pharmacology, Post-GI bleed, Anticoagulant bleeding

## Abstract

**Background:** Gastrointestinal bleeds (GIB) are associated with high morbidity and mortality, with upper GIB accounting for 20,000 deaths annually in the United States of America. Accurate risk stratification is essential in determining and differentiating high-risk *versus* low-risk patients, as low-risk patients have an overall better prognosis. Patients taking antithrombotics to reduce the risk of thromboembolic events have a 4% chance of developing a GIB. This then places physicians in a difficult position as they must perform a risk-and-benefit analysis of whether to reinstate antithrombotics after a major GIB. This systematic review aims to assess the general trends in time for resuming anticoagulation in the setting of upper GI bleed.

**Methods:** A literary search of three different databases was performed by three independent reviewers. The research databases included PubMed, ScienceDirect, and ProQuest. Specific keywords were used to narrow the search and articles were screened based on inclusion and exclusion criteria.

**Results:** Our initial search generated 11,769 potential articles and 22 articles were ultimately used for this review using specific inclusion and exclusion criteria. There is an increase in thrombotic events following a GIB if anticoagulants are not resumed. We also found that the best time to resume therapy was 15-30 days post-GIB.

**Conclusions:** Therefore, the decision to resume anticoagulation therapy should consider the patients’ medical history and should fall within 15-30 days post-GIB.

## Introduction

Gastrointestinal bleeding (GIB) is any bleeding that originates within the GI tract from the esophagus to the anus. This bleeding can be microscopic and only detected by lab testing or visible as blood in the stool or emesis. ^1^ In most cases, GIBs are classified into two broad categories: upper and lower GIBs. ^2^ Upper GIBs refers to bleeding originating from a source proximal to the Ligament of Trietz ^3^ and is usually associated with hematemesis (vomiting of blood) and melena (black, tarry stool). According to El-Tawil *et al*., Upper GI bleeds affect 50-100 out of every 100,000 Americans per year and accounts for 20,000 deaths. ^4^ Lower GIBs are classified as originating from a source distal to the Ligament of Treitz and are commonly associated with hematochezia (bright red blood in stool). ^5^ Lower GIBs are less common, accounting for approximately 20-30% of all GIBs. ^6^

### Anticoagulants and antithrombotics

Each year, there is an increasing number of patients being prescribed anti-thrombotic and anticoagulant therapies. Unfortunately, these therapies tend to place patients at an increased risk of developing GIBs. Current research indicates that 4% of individuals on anticoagulants experience GIBs at some point during treatment. ^7^ The decision to restart anticoagulation and antithrombotic therapy in patients post-GIB is a challenging task for many physicians; this makes risk stratification and shared decision making essential in the judgement to restart therapy. ^8^

*Anticoagulants mechanism*

Anticoagulants act as blood thinners by preventing the synthesis of clotting factors or by directly blocking them. Warfarin works via competitive inhibition of the enzyme vitamin K epoxide reductase to decrease the synthesis of vitamin K-dependent clotting factors II, VII, IX, X, and proteins C and S that aid in coagulation. ^9^ Prescribing warfarin to patients requires careful monitoring and assessment by physicians due to various factors, such as the patient’s genes, laboratory values, and a diet rich in vitamin K. These factors may affect the proper dosage of warfarin. ^9^ Warfarin is administered orally and inhibits multiple clotting factors, whereas direct oral anticoagulants (DOACs) work by blocking one specific clotting factor. Examples of DOACs are dabigatran, rivaroxaban, apixaban, and edoxaban. ^10^

*Antithrombotic mechanism*

Antithrombotics, such as aspirin, prohibits the aggregation of platelets in the vasculature and reduce the risk of thrombosis. ^11^ Aspirin works by permanently inhibiting COX-1 and COX-2. Aspirin as an antithrombotic drug, rather than an anti-inflammatory drug, requires a much smaller dosage to inhibit COX-1; aspirin at higher doses inhibits COX-2 and acts as an anti-inflammatory agent. ^11^ By inhibiting COX-1, arachidonic acid cannot be converted to thromboxane A _2_, a prostanoid that works to stimulate platelet aggregation. Aspirin is therefore a powerful antithrombotic drug that works to inhibit the synthesis of platelet modulators. ^11^ Other known antithrombotics work in similarly to inhibit platelet aggregation. Therefore, antithrombotics work as blood thinners and reduce the risk of thromboembolic events and ischemic stroke.

Physicians who decide to restart patients on anticoagulants must also decide the best time to do so. We sought to determine if there was a timeframe in which restarting anticoagulants and anti-thrombolytics best reduced the risk of rebleed while also protecting patients from a thrombotic event.

## Methods

A comprehensive literature search was performed using ProQuest, Science Direct, and PubMed databases from the 1 December 1992 to the 31 of December 2022. Keywords included ‘resuming anticoagulation and gastrointestinal bleeding’, ‘re-starting anticoagulation after gastrointestinal bleeding’, and ‘resuming antithrombotic after gastrointestinal bleeding’. The electronic search focused on peer-reviewed journals deemed to be in line with the goal of this paper. Articles not written in English, articles published before 1992, and duplicate articles were excluded during the screening process. Once the search was complete, three co-authors reviewed the results independently. Articles gathered from the investigation were analyzed based on their titles, study type, abstract, and full-text accessibility. Our initial search in the previously mentioned databases resulted in 11,764 articles. These selected articles were further narrowed down according to keyword specifics and preview of abstracts according to the inclusion and exclusion criteria; a total of 22 articles were deemed to be within our interest.

### Inclusion criteria

The following inclusion criteria were used: articles written in English, articles conducted on humans, articles published between 1992 and 2022, articles relevant to our interest, articles that are full-text, peer-reviewed, and include case-control, meta-analysis, observational, and cohort studies.

### Exclusion criteria

**Figure 1.**
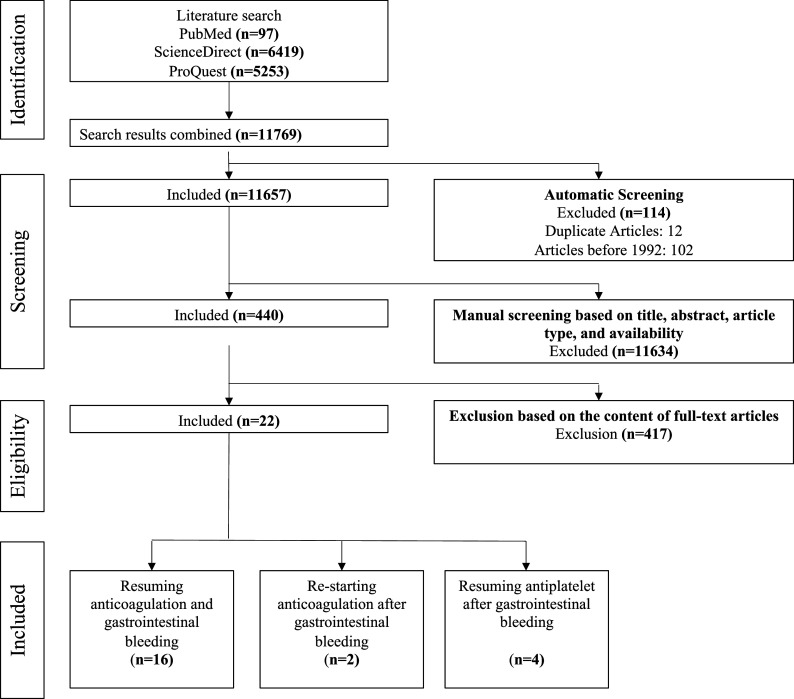
Flow of article analysis and extraction for this systematic review.

Exclusion criteria used were articles not written in English, articles published before 1992, systematic reviews, case reports, or review articles. All duplicates and non-full-text articles were also excluded. The process of inclusion and exclusion of articles is illustrated in Figure 1.

### Bias

The studies were assessed for bias. It was determined that there was a medium risk for bias as the studies were primarily conducted based on medical reports and insurance claims. The risk of bias of the individual studies were assessed using the Grading of Recommendations, Assessments, Development and Evaluation (GRADE). GRADE is a tool that evaluates flaws like imprecision, indirectness, and publications.

## Results

A total of 11,764 articles were found; 92 were from PubMed, 6419 from ScienceDirect, 5253 from ProQuest, and four were found from citations of included articles. Among the exclusions were 12 duplicate articles and 102 articles published before 1992. This resulted in 114 articles being excluded from the automatic screening process, leaving 11,657 articles for manual screening. Articles were manually screen based on the title, study type, abstract, and availability, resulting in 435 articles to be checked for eligibility. Ultimately, 22 articles were used ( Table 1).

**Table 1.** Articles on resuming/restarting anticoagulation or antithrombotic post-GIB obtained from database search.

	Author	Country	Design & study population	Findings	Conclusion
1	Qureshi *et al.*, 2014	USA	Retrospective cohort study (n = 1329)	49% of patients restarted on warfarin on average 50 days with significantly low mortality (p < 0.04). Thromboembolism event at <7 days, 7-15 days, 15-21 days, and 21-30 were as follows: HR 0.76, p = 0.47; HR 0.48, p = 0.09; HR 0.6, p = 0.14; and HR 1, p > 0.99; and GI bleed: HR 3.27, p = 0.002; HR 1.03, p = 0.93; HR 1.42, p = 0.37l and HR 1.5, p = 0.42.	Patients not started on anticoagulants had an increased risk of thromboembolism events. The risk of GIB after restarting warfarin was decreased after waiting for 1 week post-GIB.
2	Sengupta *et al.*, 2018	USA	Retrospective cohort study (n = 1338)	Restarting DOAC therapy within 30 days was not associated with thromboembolism (HR 0.98; 95% CI) or recurrent GIB (HR 1.44; 95%).	Resuming DOAC therapy was not associated with GIB recurrence or thromboembolism within 90 days.
3	Proietti *et al.*, 2018	United Kingdom	Meta-analysis (n = 5685)	Relative risk reduction of thromboembolism was significant at 46% (p < 0.00001). OAC restarters had 10.8% risk reduction for all-cause death (odds-ratio (OR) 0.38; 95% CI; p < 0.0001). Significant higher risk of recurrent major bleed (OR 1.85; 95% CI).	There’s a positive clinical benefit to restarting (OAC) compared to not restarting, with a significant reduction in thromboembolism.
4	Little *et al.*, 2019	Canada	Meta-analysis (n = 3098)	There was a reduced risk of thromboembolism (relative risk (RR) 0.30, 95% CI (9 studies)) and death (RR 0.51, 95% CI (8 studies)) and an increased risk of recurrent GIB (RR 1.91, 95% CI (11 studies)) in patients who resumed OAC.	Resuming OAC after a GIB is related to an increase in recurrent GIB while reducing the risk of thromboembolism and death.
5	Quershi and Nasir, 2017	Denmark	Cohort study (n = 3409)	Restarting anticoagulants was linked with the lowest rate of all-cause mortality (HR 0.39, 95% CI) and thromboembolism (HR 0.41), as well as an increased risk of significant bleed (HR 1.37, 95% CI).	Restarting anticoagulants post-GIB reduces the risk of thromboembolism and all-cause mortality and increases the risk of recurrent GIB.
6	Shen *et al.*, 2014	USA	Retrospective cohort study (n = 1342)	There was no increased risk of death or recurrent GIB (HR 0.94, 95% CI; HR 0.66, 95%) after resuming heparin for hemodialysis.	Resuming heparin at the first hemodialysis appointment post, GIB is not associated with an increased risk of death or re-bleed.
7	Little *et al.*, 2021	Canada	Retrospective cohort study (n = 6793)	The medium time to resumption of anticoagulants post-GIB was 46 days and was associated with a reduced risk of thromboembolism (HR 0.6; 95% CI) and mortality (HR 0.54; 95%) as well as an increased rate of rebleeding (HR 1.88; 95% CI).	Resuming OAC is linked to a reduction in thrombosis and mortality and an increase in rebleeding post GIB.
8	Witt *et al.*, 2012	USA	Retrospective cohort study (n = 442)	Warfarin resumption after GIB was associated with a reduced risk of thrombosis (HR 0.05; 95% CI) and death (HR 0.31; 95% CI) with no significant increased risk for recurrent GIB (HR 1.32; 95% CI).	Resumption of warfarin after GIB is associated with a reduced risk of thrombosis and death and will outweigh the risk of recurrent GIB.
9	Smit and Gelder, 2017	Netherlands	Retrospective cohort study (n = 1539)	All-cause mortality and risk of ischemic stroke were reduced in both dabigatran (HR 0.66; 95% CI) and warfarin (HR 0.76, 95% CI) users. Recurrent bleeding was higher in warfarin than in dabigatran patients (HR 2.31; 95% CI) and patients who discontinued anticoagulants (HR 1.56; 95% CI). There was no significant difference in recurrent bleeding between dabigatran users or those who discontinued anticoagulation.	After major bleeding, the resumption of anticoagulation therapy should be considered to reduce the risk of ischemic stroke and all-cause mortality.
10	Staerk *et al.*, 2015	Denmark	Cohort study (n = 3409)	The lowest risk of all-cause mortality (HR 0.39, 0.34 to 0.46) and thromboembolism (HR 0.41, 0.31 to 0.54) occurred with a restart of a single treatment with an oral anticoagulant.	Patients who survive the first 90 days post-GIB should restart a single treatment with an oral anticoagulant as it has the lowest risk of all-cause mortality and thromboembolism.
11	Tapaskar *et al.*, 2022	USA	Cohort study (n = 2991)	Restarting warfarin increased the risk of recurrent GIB (HR 2.12; 95% CI). Rivaroxaban was associated with recurrent GIB (HR 2.73, 95%). Warfarin (HR 0.61; 95% CI) and DOAC (HR 0.52; 95% CI) reduced the risk of thromboembolism.	Patients who restarted either warfarin or DOAC were at reduced risk of thromboembolism post-GIB. Those who restarted warfarin or rivaroxaban were at increased risk of recurrent GIB.
12	Tapaskar *et al.*, 2020	USA	Meta-analysis (n = 4376)	Restarting anticoagulation therapy increased the risk of recurrent GIB (OR 1.646; 95% CI; p = 0.035). Restarting anticoagulation therapy decreased the risk of thromboembolism (OR 0.340; 95% CI; p = 0.001). Restarting anticoagulation therapy decreased all-cause mortality (OR 0.499; 95% CI; p < 0.0001 *)*	Restarting anticoagulants post-GIB increased recurrent GIB while also decreasing thromboembolism and all-cause mortality.
13	Wang *et al.*, 2020	Taiwan	Retrospective cohort study (n = 4155)	Restarting NOAC was associated with the risk of ischemic stroke (HR 1.14; 95% CI) and recurrent bleeding (HR 1.12; 95% CI). Restarting VKA showed no significant difference in risk of ischemic stroke and recurrent bleeding.	Restarting NOAC therapy is associated with the risk of ischemic stroke and major bleeding in atrial fibrillation patients with hematuria than restarting VKA.
14	Yanagisawa *et al.*, 2021	Japan	Retrospective cohort study (n = 96)	Most patients restarted anticoagulation therapy after bleeding, and half of them continued the same drug and dosage. 90% of patients who resumed anticoagulation therapy did so within 14 days of withdrawal.	Restarting DOAC was associated with recurrent GIB, however, discontinuing anticoagulation therapy increased the risk of thromboembolism.
15	Majeed *et al.*, 2017	Sweden and Canada	Retrospective cohort study (n = 207)	58% of patients on VKA were restarted on anticoagulants at a median, interquartile range of one (0.2-3.4) week after GI bleeding event. Reduced risk of thromboembolism (HR 0.19) and death (HR 0.61). Increased risk of recurrent GI bleeding event (HR 2.5).	Rate of rebleeding after a VKA-associated upper GI bleeding event is increased however, discontinuing VKA increases the risk of thromboembolic events. Resuming VKA after a GI bleeding event is associated with a lower mortality rate, and the optimal time of resumption is between 3-6 weeks.
16	Zhou *et al.*, 2020	China	Meta-analysis (n = 12197)	Restarting OACs reduced the risk of thromboembolism (RR 0.61; 95% CI; p = 0.007). Restarting warfarin reduced the risk of thromboembolism (RR 0.59; p = 0.05). NOACs s did not reduce the risk of thromboembolism (RR 1.37 p *=* 0.18). However, they reduced the risk of all-cause mortality (RR 0.42; 95% CI; p < 0.001 *).* Restarting OACs was not associated with recurrent bleeding (RR 0.98; 95% CI p *=* 0.89).	Restarting OACs reduces the risk of thromboembolism and all-cause mortality after significant bleeding without increasing the risk of recurrent bleeding.
17	Chai-Adisaksopha *et al.*, 2015	Canada	Meta-analysis (n = 1825)	Resumption of warfarin was associated with a reduced thromboembolic event (HR 0.68;95% CI) and reduced mortality (HR.76; 95% CI p < 0.004). There was no significant increase in the risk of rebleeding (HR 1.20, 95 % CI, p = 0.10).	Resuming warfarin after GI bleeding event is associated with a 32% reduction in the risk of thromboembolism and a 24% reduction in mortality without a significant increase in rebleeding risk.
18	Hafiz *et al.*, 2021	USA	Retrospective observational cohort study (n = 57)	Thirty-four patients were on andexanet alfa, 59% of whom were restarted on either a therapeutic or prophylactic dose of andexanet alfa within 30 days of significant bleeds. Twenty-three patients were on Four-Factor Prothrombin Complex Concentrate (4F-PCC), 65% were restarted on 4F-PCC within 30 days. The median time for resuming anticoagulants was three days.	The timing of reinitiating anticoagulants after a significant bleeding event may differ based on the clinical presentation and severity of bleeding.
19	Valanejad *et al.*, 2020	USA	Retrospective single system study (n = 57)	Recurrent GIB was 5.6% and 2.5% in patients who resumed and did not resume their DOAC respectively.	Patients who resumed anticoagulants within seven days post GIB were not associated with a rebleed within 90 days of discharge.
20	Sostres *et al.*, 2019	Spain	Observational Cohort Study (n = 871)	80.5% of patients resumed anticoagulants or antithrombotic 7.6 ± 36.4 days following a gastrointestinal bleed. Resumption before the 7th day showed no significant difference in mortality. 98.5% of patients resumed anticoagulants or antithrombotic within 30 days. Resumption of therapy was associated with a higher risk of re-bleeding (HR 2.184; 95% CI) but a lower risk of ischemic events (HR 0.626; 95% CI). Patients taking both anticoagulants and antithrombotic had a lower mortality rate than those on a single therapy (15.9% *versus* 29.3%; p = 0.009).	Resuming anticoagulants or antithrombotic after a Gastrointestinal bleeding event has been associated with a lower risk of vascular events and a higher risk of rebleeding; however, the benefits of reinstating these therapies far outweigh the risk of rebleeding. Resuming therapy after one week is overall beneficial to the patient.
21	Lee *et al.*, 2011	South Korea	Retrospective Study (n = 58)	Rebleeding occurred in 7% of patients on warfarin and 0% of patients on aspirin (p = 0.03). 16.7% of patients on warfarin and 2.4% of those on aspirin had a thromboembolic event (p < 0.01). Mortality was 3.4% in warfarin users, and none was noted in aspirin users.	Individual patients’ clinical presentations should be taken into consideration. However, it is recommended that Anticoagulants be resumed by the 20th day to prevent thromboembolic events.
22	Candeloro *et al.*, 2021	Italy	Retrospective cohort study (n = 948)	Anticoagulants resumption results in an increase of recurrent GIB bleed (HR 1.5, 95% CI) but a lower risk of a thromboembolic event (HR 0.34, 95% CI) and death (HR 0.50, 95% CI).	There was an increased risk of recurrent bleeding in patients restarted on anticoagulants but also a reduced risk of thrombosis and mortality.

GIB: GI bleed; HR: hazard ratio; CI: confidence interval; DOAC: direct oral anticoagulant; OAC: oral anticoagulant; NOAC: novel oral anti-coagulant; VKA: vitamin K antagonist; OR: odds-ratio.

Overall, 18 articles focused on resuming anticoagulant therapy post-GIB and the major findings presented indicated that those who resumed therapy had a reduced risk of a thrombotic event. There were some patients who did have a rebleed but the overall outcomes showed that the benefits from resuming anticoagulant therapy outweighed the risk. With respect to the four antithrombotic/antiplatelet therapy articles, there were similar results; however there was a great risk in rebleed as most of these therapies are given in conjunction with anticoagulants.

## Discussion

### Oral anticoagulants

The decision to resume anticoagulants and antithrombotic post-GIB is crucial when confronted with patients with a history of thrombotic events, mechanical heart valves, and atrial fibrillation, despite the associated risk of recurrent bleeding. ^12^ The clinical impacts of thromboembolism and recurrent GIB are not equivalent and need to be accounted for when determining whether to resume anticoagulant and antithrombotic therapy. Little *et al*. reports that the case-fatality rate and institutionalization due to a thromboembolic event at three months post-GIB was 41% (Europe) and 57% (U.S.). ^12^ In contrast, the case-fatality rate of oral anticoagulants (OAC)-caused recurrent GIB was between 8-13%. ^12^ Research has shown that while there is an increase in the risk of recurrent bleeding, there is a decreased risk of thrombolytic events and all-cause mortality.

According to multiple studies, an average of 60% of patients who suffered a GIB were resumed on OACs as a preventive measure. ^12^^,^^13^ Up to half of the patients resuming OACs post-GIB were restarted on the same drug and dosage, and around 40% of patients were switched to a drug that was not an OAC. ^14^ Restarting OACs in patients who suffered atrial fibrillation and bleeding events were associated with a significant decrease in thromboembolism and did not significantly increase the risk of recurrent bleeding. This then contributed to a reduction of all-cause mortality in patients. ^13^^,^^15^^–^^18^ Proietti *et al.*, found a decreased risk of thromboembolic events in patients who restarted OACs *versus.* those who did not, with a relative risk reduction of 44%. ^19^ There was also a significant reduction in all-cause mortality and thromboembolic event (hazard ratio (HR) 0.35; 95% confidence interval (CI); HR 0.76; 95% CI) in patients who were restarted on OACs. ^19^

Sostres *et al.*, compared patients who resumed OACs *versus* those who did not after a GIB, they found that of the 811 patients that stopped therapy, 17.8% of patients experienced a thromboembolic event during the follow-up period, while 24.9% had a recurrent GIB. ^20^ There was a significant difference in those that resumed therapy *versus* those who did not, with patients who resumed having a lower risk of an ischemic event (HR 0.626; 95% CI) and a decreased mortality (HR.0606), although they did have a higher risk of rebleeding (HR 2.184; 95% CI). ^20^ Rebleeding during the time following the initial GIB is a likely event. Candeloro *et al.* explored the status of recurrent GIB in patients who restarted and discontinued OACs post GIB and found that the rebleeds tend to occur where the original bleed stemmed from Ref. 18. Proietti *et al.*, found that recurrent GIBs occurred in 10.2% of patients who resumed OACs compared to 5% of patients who did not restart. ^19^ Similarly, Tapaskar *et al.*, found that 10.1% of patients who resumed OACs post-GIB had recurrent GIB compared to 5.3% of patients who discontinued OACs. ^21^ Across those studies, there was an average of a 5% increase in risk for GI bleed; both studies found these results insignificant.

### Warfarin

Several studies have been conducted to assess the resumption of warfarin post-GIB, as it is infamously known for its unpredictability. Witt *et al.*, found that at least 35% of patients resumed warfarin after a GIB. ^22^ Smit and Gelder showed that after significant bleeding, 47% of warfarin users were restarted on their therapy, and there was a decrease in thromboembolic events and all-cause mortality. In a study population of 1825, Chai-Adisaksopha *et al.* reported a significant reduction in thromboembolic events in patients who resumed warfarin therapy post-GIB. ^23^ Across multiple studies, there was an average of 6% decrease in thrombolytic events for those who resumed warfarin therapy. ^12^^,^^22^^–^^24^ Resumption of warfarin was associated with a significant reduction in all-cause mortality in patients. ^22^^–^^26^ Chai-Adisaksopha *et al.* reported that death occurred in 24.6% of patients who resumed warfarin, whereas, in patients who did not resume warfarin, death occurred in 39.2% of patients. ^23^ Similarly, Little *et al.* observed that the all-cause mortality rate was 21.5% in patients who resumed warfarin compared to 31.6% who did not. ^12^

Across all studies, only one patient death was reported which was associated with a massive gastrointestinal bleed in a patient who resumed warfarin. ^25^ On average, rebleeding occurred in 11.6% of patients restarted on warfarin and 5.7% of patients who were not restarted; however, the studies found that these differences were not statistically significant. ^17^^,^^19^^,^^22^^–^^24^^,^^26^^,^^27^ Although it is assumed that patients who do not resume anticoagulants are at a lower risk of rebleeding and bleeding-related complications, Majeed *et al.* demonstrated that these patients would require closer monitoring and shorter follow-up (92 weeks) as compared to those who were restarted on VKAs (142 weeks, p < 0.001). ^26^ The rate of recurrent bleed is high whether the patient is resumed on anticoagulants or not, with bleeding occurring at a median of 24 weeks in those that were resumed on anticoagulants and 23 weeks in patients that were not. ^26^

### Direct oral anticoagulants

Some physicians decide to switch treatment plans, such as changing from warfarin to DOAC post-GIB. ^18^^,^^28^ Little *et al.* found that apixaban was the DOAC of choice in most cases. ^12^ There was a 3% increase in patients who experienced a recurrent GIB after the resumption of DOACs. ^12^^,^^27^ Proietti *et al.* focused on dabigatran and found that patients who were started on dabigatran had a reduced risk for recurrent GIB compared to those who started on warfarin. ^18^ Smit and Gelder reported that 49% of dabigatran users restarted anticoagulation after significant bleeding, and the risk of ischemic stroke and all-cause mortality decreased. ^29^ There was some conflict on the overall effectiveness of DOAC’s competence post-GIBs. Sengupta *et al.* found that patients with a history of venous thromboembolism who were restarted on DOAC had a risk of 2.7% for a thromboembolic event compared to 2.2% in patients whose DOAC was held 90 days post-GIB. ^30^ In that same year, however, Proietti *et al.* found a reduced risk of thromboembolic events and all-cause mortality in patients who restarted on DOACs after a GIB. ^19^

Patients who restarted on DOACs within 30 days of the index bleed were not associated with a recurrent bleed. ^30^ Sengupta *et al.* reported no difference in readmission for a recurrent GIB in patients started on DOACs compared to those who were not restarted on DOACs. ^30^ Other studies, however, have reported recurrent GIB issues associated with rivaroxaban. ^17^^,^^27^^,^^30^ Valanejad *et al.* demonstrated that rivaroxaban was the only DOAC that increased the risk of recurrent GIB. ^27^ Overall, most research reports that the resumption of DOACs reduces the risk of thromboembolism and all-cause mortality with a selective increase in recurrent GIBs. ^12^^,^^17^^,^^19^^,^^28^ The results were relatively similar when comparing DOACs with warfarin use, but a benefit of the DOACs with resuming treatment post-GIB is the predictability and reduced monitoring that is typically associated with DOACs as compared to warfarin.

### Heparin

Heparin is a low molecular weight anti-coagulant typically used as a bridging therapy. Individuals with a history of thrombolytic events, cancer, and who were younger tended to be restarted on heparin post-GIB by the first outpatient hemodialysis session. ^31^ Heparin doses tend to be decreased post-GIB, and Shen et. al found no significant association between recurrent GIB (HR.78, 95% CI) and death (HR 1.01, 95%) compared to those who did not resume heparin therapy [39]. Resumption of heparin in both studies reduced thrombolytic events while non-significantly contributing to recurrent GIB. ^18^^,^^31^

### Antithrombotic therapy

Antithrombotic therapy resumption post-GIB was also a point of interest. Studies showed that antithrombotic were paired with anti-coagulants and proton-pump inhibitors and were typically not given alone. ^16^^,^^18^^,^^20^^,^^21^^,^^30^^,^^32^ Of the patients on antithrombotics, 8.5% were discontinued to reduce rebleeding risk, while 6.8% of patients were switched to a different antithrombotic. ^18^ Patients restarted on antithrombotics with anticoagulants, and a small few alone, had a higher risk of rebleed than those just resuming OAC. ^16^ Patients on dual antithrombotic therapy were also associated with lower mortality (15.9%) than those that were not (29.3%; p = 0.009). ^20^ Sostres *et al.*, also reported that patients who did not resume anticoagulant and/or antithrombotic therapy within 90 days after the initial bleed had higher all-cause mortality and higher risk of thromboembolism. ^20^ Tapaskar *et al.*, however, reported that they were unable to identify the significance of resuming or discontinuing antithrombotic in patients post-GIB. ^21^ Sengupta *et al.* did discover that patients who were taking thienopyridines were more likely to have a recurrent GIB. ^30^ Ultimately, anticoagulants were better than antithrombotic in terms of thromboembolic and mortality outcomes with less incidences of recurrent bleeds.

### Timing to resume anticoagulants

Studies have noted that resuming anticoagulation is optimal around 90 days. However, other studies have indicated that thrombotic events occur within these days, leaving with a greater increase in mortality. ^16^^,^^20^^,^^27^^,^^33^ Narrowing down this time frame is optimal. The type and severity of the GIB plays a role in the clinician’s decision on when to resume anticoagulants. ^33^ Hafiz *et al.* suggested a prophylactic dose of anticoagulants be administered before graduating to a full dose to balance the risk of a thromboembolic event. ^33^ They also indicated that resuming antithrombotic therapy within seven days of the initial bleeding event has an overall beneficial effect. ^33^ Sostres *et al.* found that 98.5% of patients reinstated therapy within the first 30 days (median of 6 and mean of 7.6 ± 6.4 days), suggesting that the first week might be the optimal time for resuming anticoagulant or antithrombotic therapy. ^20^ However, they also stress that clinicians should use risk stratification tools and make decisions case-by-case. ^20^

Other studies have shown that as compared to patients that were resumed on therapy at least seven days after the index bleed, patients that were resumed on therapy within the first seven days were associated with a higher rate of rebleeding (30.6% p = 0.044) and a lower rate of ischemic events (13.6%; p = 0.025). ^20^^,^^22^^,^^25^ Patients who resumed after day 7 had a lower rebleeding rate than those who restarted anticoagulants within the first seven days. ^25^ Mortality during follow-up of patients was lowest when warfarin was restarted between 15-90 days post-GIB. ^22^ Lee *et al.* suggests the optimal timing for resumption of warfarin lies between 14-20 days after the initial bleeding event after a SEE has been performed to ensure that the patient is hemodynamically stable. ^24^ Majed *et al.* however, suggested that the risk of rebleeding within the first three weeks after the index bleed is very high and that risk reduces significantly by the sixth week. Hence, the optimal time for resumption lies between the third and sixth week after the initial bleed. ^26^ The time frame varies greatly between <7 days up to 90 days, ultimately showing a decrease in thrombolytic events. Most research points toward 15-30 days post GIB as an optimal window as to when to resume therapy, but it is still clear that this needs to be assessed based on the patient’s presentation and medical history.

### Limitations to resuming anticoagulants and antithrombotic therapies

While we found a greater benefit in resuming anticoagulant and antithrombotic therapies post-GIB, there are still factors to consider as to why physicians wouldn’t resume these therapies. Physicians tend to hesitate resumption in patients with upper, lower, and unknown-source GIB, diabetes, renal disease, coronary artery disease, and history of falls, specifically for warfarin due to either medication adherence or increase chance in rebleeds and other adverse events. ^27^^,^^34^ Older patients or patients with dementia and living in a long-term care facility were also less likely to be placed on OACs post GIB. ^24^ Patients who underwent blood transfusion during hospitalization and required monitoring in intensive care units and previous GIB bleeds, were usually given alternate medication to prevent thromboembolism. ^19^^,^^30^ Quershi *et al.* reported that on top of the previously mentioned factors, 19% of patients could not follow-up treatment with an anticoagulant clinic and had insurance issues. ^25^ The physician’s preference was also indicated in 18% of the cases not to restart anticoagulation. ^25^ While this paper addresses some of the reasons for resuming anticoagulant and antithrombotic therapy, specific reasons should be addressed in further research.

### Limitations to the study

The studies did present with some limitations in that they were largely retrospective investigations using health insurance claims. Retrieving reports from health insurance claims do run the risk of being inaccurate. This also limits the study population, depending on the origin of the study, due to patient who are not covered under insurance, as their population could be an important factor to consider in rates of rebleeds or thrombolytic events.

## Conclusions

OACs significantly reduced thromboembolic and all-cause mortality rates while also increasing the risk for a recurrent GIB. Warfarin is associated with a slightly higher risk of GIB when compared to DOACs and has a less predictable outcome as a vitamin K antagonist. DOACs tend to provide the same benefits in reducing thromboembolic events and all-cause mortality as warfarin but have a lower increased risk of recurrent bleeding. Heparin resumption has also been analyzed as it is typically used for bridging therapy and for hemodialysis patients, also showing similar beneficial effects as warfarin and DOACs, with few side effects following the first outpatient session of hemodialysis as another form of anti-coagulants used to resume other treatment plans for specific conditions.

Antithrombotic also needs to be addressed following a GIB. Studies we have found showed that, while usually paired with anticoagulants, certain antithrombotic further increases the risk for recurrent bleeds. We also attempted to address the timing of when to resume anticoagulant and antithrombotic therapy, and while the research varied in resumption time, the studies indicated that an average of 15-30 days post-GIB would be the most beneficial time. The most critical factor to consider when resuming anticoagulation and antithrombotic therapy, however, is the patient’s medical history such as atrial fibrillation, dementia, renal disease, among others. Some patients are unable to safely resume therapy due to deteriorating cognition or inability to receive the appropriate care. However, physicians’ future decisions should consider that the benefits outlined here may be greater than the risk of recurrent GIB, especially when timed appropriately.

Ultimately through our search, we found there is a benefit from using antithrombotics post-GIB. Most of these benefits occur through reducing the possibility of thrombolytic events such as pulmonary embolism or stroke. There are more positive results using a single anticoagulant with slight differences between warfarin of DOACs, both reducing the risk of a thrombolytic event. Differences between each is the risk in recurrence of a rebleed; however, when compared to a clot, the rebleed is not enough to outweigh the benefits of preventing this sort of events. Antithrombotics have also be reviewed, and while they’re usually taken with an OAC, there is a greater risk of rebleed compared to with OACs alone.

## Data Availability

All underlying data are included as part of the article and no additional data are required. Slouha, E., Jensen, H., Fozo, H., Raj, R., Thomas, S., & Gorantla, V. (2023). Re-starting Anticoagulation and Antiplatelets after Gastrointestinal bleeding: A Systematic Review (Version 1). Figshare: PRISMA checklist for “Re-starting Anticoagulation and Antiplatelets after Gastrointestinal bleeding: A Systematic Review”, https://doi.org/10.6084/m9.figshare.22722526.v1 . ^35^ Data are available under the terms of the Creative Commons Zero “No rights reserved” data waiver (CC0 1.0 Public domain dedication).

## References

[1] Zhao Y Encinosa W : *Hospitalizations for Gastrointestinal Bleeding in 1998 and 2006. HCUP Statistical Brief #65.* Rockville, MD: Agency for Healthcare Research and Quality;December, 2008. Reference Source 21595135

[2] *Gastrointestinal bleeding.* UCLA Health System. n.d.Retrieved October 25, 2022. Reference Source

[3] Antunes C Copelin EL II : Upper Gastrointestinal Bleeding. [Updated 2021 Jul 21]. *StatPearls.* Treasure Island (FL): StatPearls Publishing;2022 Jan. Reference Source

[4] El-Tawil AM : Trends on gastrointestinal bleeding and mortality: where are we standing? *World J. Gastroenterol.* 2012;18(11):1154–1158. 10.3748/wjg.v18.i11.1154 22468077 PMC3309903

[5] Ghassemi KA Jensen DM : Lower GI bleeding: epidemiology and management. *Curr. Gastroenterol. Rep.* 2013;15(7):333. 10.1007/s11894-013-0333-5 23737154 PMC3857214

[6] Amin SK Antunes C : Lower Gastrointestinal Bleeding. [Updated 2021 Jul 19]. *StatPearls.* Treasure Island (FL): StatPearls Publishing;2022 Jan. Reference Source 28846221

[7] Testa S Ageno W Antonucci E : Management of major bleeding and outcomes in patients treated with direct oral anticoagulants: results from the START-Event registry. *Intern. Emerg. Med.* 2018;13(7):1051–1058. 10.1007/s11739-018-1877-z 29790125

[8] Smit MD Van Gelder IC : Resumption of anticoagulation after major bleeding decreases the risk of stroke in patients with atrial fibrillation. *Evid. Based Med.* 2017;22(3):107–108. 10.1136/ebmed-2017-110694 28512109

[9] Patel S Singh R Preuss CV : Warfarin. [Updated 2022 Jan 19]. *StatPearls.* Treasure Island (FL): StatPearls Publishing;2022 Jan. Reference Source

[10] Vazquez S Rondina MT : Direct oral anticoagulants (DOACs). *Vasc. Med (London, England).* 2015;20(6):575–577. 10.1177/1358863X15600256 26285587

[11] Eikelboom JW Hirsh J Spencer FA : Antiplatelet drugs: Antithrombotic Therapy and Prevention of Thrombosis, 9th ed: American College of Chest Physicians Evidence-Based Clinical Practice Guidelines. *Chest.* 2012;141(2 Suppl):e89S–e119S. 10.1378/chest.11-2293 22315278 PMC3278069

[12] Little D Chai-Adisaksopha C Hillis C : Resumption of anticoagulant therapy after anticoagulant-related gastrointestinal bleeding: A systematic review and meta-analysis. *Thromb. Res.* 2019;175:102–109. 10.1016/j.thromres.2019.01.020 30743134

[13] Wang CL Wu VC Huang YT : Incidence and consequences of resuming oral anticoagulant therapy following hematuria and risks of ischemic stroke and major bleeding in patients with atrial fibrillation. *J. Thromb. Thrombolysis.* 2021;51(1):58–66. 10.1007/s11239-020-02135-2 32409936

[14] Yanagisawa D Abe K Amano H : Thrombotic events and rebleeding after hemorrhage in patients taking direct oral anticoagulants for non-valvular atrial fibrillation. *PLoS One.* 2021;16(11):e0260585. 10.1371/journal.pone.0260585 34843582 PMC8629319

[15] Zhou Y Guo Y Liu D : Restarting of anticoagulation in patients with atrial fibrillation after major bleeding: A meta-analysis. *J. Clin. Pharm. Ther.* 2020;45(4):591–601. 10.1111/jcpt.13130 32181518

[16] Staerk L Lip GY Olesen JB : Stroke and recurrent haemorrhage associated with antithrombotic treatment after gastrointestinal bleeding in patients with atrial fibrillation: nationwide cohort study. *BMJ (Clinical Research ed.).* 2015;351:h5876. 10.1136/bmj.h5876 26572685 PMC4646074

[17] Tapaskar N Pang A Werner DA : Resuming Anticoagulation Following Hospitalization for Gastrointestinal Bleeding Is Associated with Reduced Thromboembolic Events and Improved Mortality: Results from a Systematic Review and Meta-Analysis. *Dig. Dis. Sci.* 2021;66(2):554–566. 10.1007/s10620-020-06248-9 32279174

[18] Candeloro M Es N van Cantor N : Recurrent bleeding and thrombotic events after resumption of oral anticoagulants following gastrointestinal bleeding: Communication from the ISTH SSC Subcommittee on Control of Anticoagulation. *J. Thromb. Haemost.* 2021;19(10):2618–2628. 10.1111/jth.15476 34318606

[19] Proietti M Romiti GF Romanazzi I : Restarting oral anticoagulant therapy after major bleeding in atrial fibrillation: A systematic review and meta-analysis. *Int. J. Cardiol.* 2018;261:84–91. 10.1016/j.ijcard.2018.03.053 29572080

[20] Sostres C Marcén B Laredo V : Risk of rebleeding, vascular events and death after gastrointestinal bleeding in anticoagulant and/or antiplatelet users. *Aliment. Pharmacol. Ther.* 2019;50(8):919–929. 10.1111/apt.15441 31486121

[21] Tapaskar N Ham SA Micic D : Restarting Warfarin *versus* Direct Oral Anticoagulants After Major Gastrointestinal Bleeding and Associated Outcomes in Atrial Fibrillation: A Cohort Study. *Clin. Gastroenterol. Hepatol.* 2022;20(2):381–389.e9. 10.1016/j.cgh.2020.11.029 33227428

[22] Witt DM Delate T Garcia DA : Risk of thromboembolism, recurrent hemorrhage, and death after warfarin therapy interruption for gastrointestinal tract bleeding. *Arch. Intern. Med.* 2012;172(19):1484–1491. 10.1001/archinternmed.2012.4261 22987143

[23] Chai-Adisaksopha C Hillis C Monreal M : Thromboembolic events, recurrent bleeding and mortality after resuming anticoagulant following gastrointestinal bleeding. A meta-analysis. *Thromb. Haemost.* 2015;114(4):819–825. 10.1160/TH15-01-0063 26018236

[24] Lee JK Kang HW Kim SG : Risks related with withholding and resuming anticoagulation in patients with non-variceal upper gastrointestinal bleeding while on warfarin therapy. *Int. J. Clin. Pract.* 2012;66(1):64–68. 10.1111/j.1742-1241.2011.02827.x 22171905

[25] Qureshi W Mittal C Patsias I : Restarting anticoagulation and outcomes after major gastrointestinal bleeding in atrial fibrillation. *Am. J. Cardiol.* 2014;113(4):662–668. 10.1016/j.amjcard.2013.10.044 24355310

[26] Majeed A Wallvik N Eriksson J : Optimal timing of vitamin K antagonist resumption after upper gastrointestinal bleeding. A risk modelling analysis. *Thromb. Haemost.* 2017;117(3):491–499. 10.1160/TH16-07-0498 28004062

[27] Valanejad SM Davis KA Nisly SA : Outcomes Associated With Resuming Direct Oral Anticoagulant Therapy Following Admission for a Gastrointestinal Bleed. *Ann. Pharmacother.* 2020;54(10):975–980. 10.1177/1060028020912429 32141301

[28] Little D Sutradhar R Cerasuolo JO : Rates of rebleeding, thrombosis and mortality associated with resumption of anticoagulant therapy after anticoagulant-related bleeding. *CMAJ.* 2021;193(9):E304–E309. 10.1503/cmaj.201433 33649169 PMC8034308

[29] Smit MD Van Gelder IC : Resumption of anticoagulation after major bleeding decreases the risk of stroke in patients with atrial fibrillation. *Evid. Based Med.* 2017;22(3):107–108. 10.1136/ebmed-2017-110694 28512109

[30] Sengupta N Marshall AL Jones BA : Rebleeding *versus* Thromboembolism After Hospitalization for Gastrointestinal Bleeding in Patients on Direct Oral Anticoagulants. *Clin. Gastroenterol. Hepatol.* 2018;16(12):1893–1900.e2. 10.1016/j.cgh.2018.05.005 29775794

[31] Shen JI Mitani AA Winkelmayer WC : Heparin use in hemodialysis patients following gastrointestinal bleeding. *Am. J. Nephrol.* 2014;40(4):300–307. 10.1159/000367901 25341418 PMC4246045

[32] Qureshi WT Nasir U : Restarting oral anticoagulation among patients with atrial fibrillation with gastrointestinal bleeding was associated with lower risk of all-cause mortality and thromboembolism. *Evid. Based Med.* 2016;21(4):152. 10.1136/ebmed-2016-110444 27190077

[33] Hafiz A Abdulrahman IA Sylvester KW : Evaluation of anticoagulation re-initiation practices following reversal of factor Xa inhibitors with andexan *et al*fa or 4F-PCC in patients with major bleeding events. *Thrombosis Update.* 2021;5:100076. 10.1016/j.tru.2021.100076

[34] Qureshi WT Nasir U : Restarting oral anticoagulation among patients with atrial fibrillation with gastrointestinal bleeding was associated with lower risk of all-cause mortality and thromboembolism. *Evid. Based Med.* 2016;21(4):152. 10.1136/ebmed-2016-110444 27190077

[35] Slouha E Jensen H Fozo H : Re-starting Anticoagulation and Antiplatelets after Gastrointestinal bleeding: A Systematic Review. figshare. *Journal Contribution.* 2023. 10.6084/m9.figshare.22722526.v1 PMC11222779 38966192

