# Supplementary material for: Re-starting anticoagulation and antiplatelets after gastrointestinal bleeding: A systematic review
Source: F1000Res. 2023 Jul 10;12:806. [Version 1] doi: 10.12688/f1000research.135132.1 (PMC11222779; doi:10.12688/f1000research.135132.1)
Supplement: Peer review history

# Reviewer report: Reviewer response for version 1

**DOI:** 10.5256/f1000research.148235.r292646

**Published:** 2024-07-03

**Authors:** Sudhakar Pemminati

**Affiliations:** 1 California Health Sciences University- Clovis, California, USA

I strongly recommend this article for indexing.

 The authors made special effort to review the re-starting anti-coagulation and anti-platelets after gastrointestinal bleeding.

 Gastrointestinal bleeding is one of the most important factor for morbidity and mortality in various patient population. I would like to ask authors to elaborate materials and methods.

 Thank you

Are the rationale for, and objectives of, the Systematic Review clearly stated?

Yes

Is the statistical analysis and its interpretation appropriate?

Not applicable

If this is a Living Systematic Review, is the ‘living’ method appropriate and is the search schedule clearly defined and justified? (‘Living Systematic Review’ or a variation of this term should be included in the title.)

Yes

Are sufficient details of the methods and analysis provided to allow replication by others?

Yes

Are the conclusions drawn adequately supported by the results presented in the review?

Yes

Reviewer Expertise:

Endocrine disorders

I confirm that I have read this submission and believe that I have an appropriate level of expertise to confirm that it is of an acceptable scientific standard.

# Reviewer report: Reviewer response for version 1

**DOI:** 10.5256/f1000research.148235.r226018

**Published:** 2023-12-20

**Authors:** Xue Xiao

**Affiliations:** 1 Sichuan University, Chengdu, China

1. Section of Introduction: GI appears for the first time, abbreviations should not be used, so please ask the author to modify it.

 2. Section of Introduction: May I ask if the author agrees to change"Upper GI bleeds affect 50-100 out of every 100,000 Americans per year and accounts for 20,000 deaths" to "Upper GI bleeds affect 50-100 out of every 100,000 Americans and accounts for 20,000 deaths  per year"?

 3. Ask the author if they use "re-starting anticoagulation after gastrointestinal bleeding" as a keyword for search?

 4.Section of Inclusion criteria: May I ask if the author agrees to change"articles that are full-text" to "articles that are with full-text"?

 5. Section of Heparin:Ask the author if there are some articles about used Low molecular weight heparin in such patients?If so, please add.

 6. As the author's opinion:there was a great risk in rebleed as most of these therapies are given in conjunction with anticoagulants, but there is on discussion about this in Section of  Discussion,If there is any relevant content, please ask the author to supplement it.

Are the rationale for, and objectives of, the Systematic Review clearly stated?

Yes

Is the statistical analysis and its interpretation appropriate?

Not applicable

Are sufficient details of the methods and analysis provided to allow replication by others?

Yes

Are the conclusions drawn adequately supported by the results presented in the review?

Yes

Reviewer Expertise:

This article provides guidance and suggestions for restarting anticoagulation plans in the future. However, as gynecological oncologists, we hope that more articles related to postoperative restart of anticoagulation in gynecological malignant tumor patients will be included in this study in the future.

I confirm that I have read this submission and believe that I have an appropriate level of expertise to confirm that it is of an acceptable scientific standard.
